# Silver Nitrate and Bioactive Glass Containing Chitosan Coatings: Comparison of Antimicrobial and Antiviral Properties

**DOI:** 10.1021/acsami.6c00215

**Published:** 2026-02-18

**Authors:** Zoya Hadzhieva, Elisa Feyles, Muhammad Asim Akhtar, Manuela Donalisio, Domiziana Porporato, David Lembo, Borja Coto, Sofia Alves, Olatz Areitioaurtena, Samuele Gentile, Aldo R. Boccaccini

**Affiliations:** † Institute of Biomaterials, Department of Materials Science and Engineering, University of Erlangen-Nuremberg 91058 Erlangen, Germany; ‡ Department of Clinical and Biological Sciences, Laboratory of Molecular Virology and Antiviral Research, 9314 University of Turin, Regione Gonzole 10 10043 Orbassano, Italy; § National PhD Programme in One Health Approaches to Infectious Diseases and Life Science Research, Department of Public Health, Experimental and Forensic Medicine, University of Pavia, Pavia 27100, Italy; ∥ Tekniker, Basque Research and Technology Alliance (BRTA), C/Iñaki Goenaga 5 20600 Eibar, Spain; ⊥ GV Filtri Industriali, Baldissero Torinese (To) 10020, Italy

**Keywords:** electrophoretic deposition, antibacterial, antiviral, chitosan, silver, bioactive glass, filter, coating

## Abstract

Pathogenic air and water contaminations present major health issues nowadays. Filter-based protective devices have been commonly utilized to capture airborne or waterborne microorganisms and viruses in order to reduce or inhibit the transmission of infectious diseases. Some approaches to extend the functionality of filtering devices and to further enhance their capability to inactive pathogens include the application of antimicrobial coatings on the filter surface. In this regard, the current work aims to develop innovative coatings with antiviral and antibacterial properties composed of a chitosan matrix incorporating bioactive glass (BG) nanoparticles or silver nitrate (AgNO_3_) on metallic filters by using electrophoretic deposition. The so-produced chitosan/BG- and chitosan/AgNO_3_-coated filters were characterized in terms of their morphology, chemical composition, filtering capability, antibacterial properties against *Staphylococcus aureus*, and antiviral activity against coronavirus OC43, rhinovirus, adenovirus, and influenza virus. The results confirmed that chitosan/AgNO_3_ and chitosan/BG coatings exhibited no blockage of the filter pores. The bubble point values for both coating types were similar to the measurements of uncoated filters, suggesting intact filtration capability after the application of the coatings. The antibacterial assay showed the antibacterial activity of >99% for both chitosan/AgNO_3_ and chitosan/BG coatings against *S. aureus*. Finally, chitosan/AgNO_3_-coated filters showed antiviral activity against three out of the four tested viruses (HCoV-OC43, HRV-A1, and IFV-AH3N2) and exerted a partial antiviral action against AdV-5. On the other hand, chitosan/BG-coated filters were active against all tested respiratory viruses. The outcomes of this study evidenced that the developed multimaterial coatings could serve as a suitable platform to produce antimicrobial filtering systems for a broad-spectrum application.

## Graphical abstract

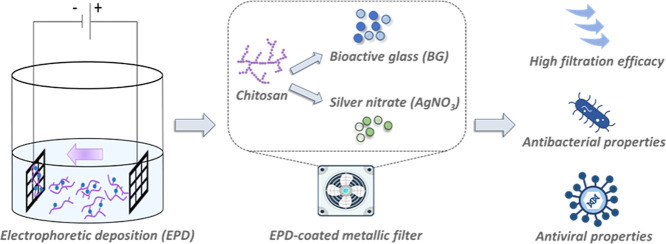

## 1 Introduction

Contaminated air, comprising particulate matter, ozone, volatile organic compounds, heavy metallic oxides, or biological contaminants, such as bacteria, viruses, fungi, mites, or parasites, is a major health issue affecting millions of people worldwide.^1,2^ The main sources of air contamination include industrial activity, transport vehicles, construction materials and equipment, plant-derived compounds (pollen, odors, allergens), etc. Among the various airborne pathogens, viruses, bacteria, and fungi pose a severe threat to human health as they may trigger a range of illnesses, including allergic reactions (hypersensitivity, allergic rhinitis, pneumonitis, asthma, etc.) and infectious diseases (influenza, common cold, pneumonia, conjunctivitis, etc.).^2^

Mechanical air filtration is widely utilized to capture contaminants due to its cost-efficiency and versatility. According to the fundamental filtration theory, there are five main filtration mechanisms for particle capture, including diffusion, interception, impaction, gravitational settling, and electrostatic attraction.^3^ One of the main complications when porous membranes are used as filter materials is the occurrence of fouling effects due to stuck or adherend contaminants within the filtering mesh. Thus, novel strategies to overcome this issue and effectively combat pathogens include integrating advanced intrinsically antiviral materials into filtration devices by applying bactericidal functional surface coatings. Owing to its broad range of beneficial characteristics, such as the simple equipment, the possibility to be applied on various 3-dimensional substrates with complex geometries, and the opportunity to adjust the desirable dense packing of particles in the final deposit, electrophoretic deposition (EPD) appears as a promising coating technique for filtration devices.^4^ Chitosan, the deacetylated form of chitin, is the most widely utilized polymer to produce antimicrobial coatings by EPD.^5^ It is a biodegradable and nontoxic polymer with a wide range of antimicrobial properties against bacteria, viruses, and fungi.^6^ In particular, the antibacterial activity of chitosan-based materials against *Bacillus subtilis*, *Escherichia coli*, *S. aureus*, *Pseudomonas aeruginosa*, *Salmonella typhimurium*, *Vibrio cholerae*, *Lactobacillus plantarum*, *L. bulgaricus*, *L. brev1parahemolyticus*, etc. has been reported previously.^7,8^ Additionally, chitosan derivatives are reported to exhibit inhibitory effects against bacteriophages (T2, T7, 1–97A, MS2, and phiX17), animal viruses (murine norovirus, feline calicivirus, Newcastle virus), plant viruses (tobacco mosaic virus), and human viruses (cytomegalovirus strain AD169, H1N1 influenza A virus, Rift Valley Fever virus (RVFV), Herpes Simplex-1 (HSV-1), Coxsackie viruses, SARS-CoV-2).^7–9^ Being a natural polysaccharide, chitosan could offer highly selective pollutant and biological filtration due to the presence of functional groups in its backbone, such as hydroxyl or amino groups, potentially attracting the contaminants.^10^ Additionally, owing to its metal ion chelation capability, chitosan has been used to attract heavy metals for chemical waste detoxification, or removal of dyes.^11^ Currently, the most common application of chitosan in filtration technology is in the production of porous filters made from chitosan nonwoven fibers or in the chitosan-containing coating of textile filters.^6,12–14^ Still, there is no published research on the application of chitosan-based coatings on metallic filters with antiviral potential. Chitosan has been previously combined with other antimicrobial agents, such as bioactive glasses (BGs) or silver particles, for biomedical coating applications.^5^ BGs are a group of nontoxic bioceramic materials with antibacterial properties originating from the local pH and osmolarity increase due to ion dissolution processes.^15^ Silver nitrate (AgNO_3_) is another potent antimicrobial agent, which has been historically recognized for applications in disinfecting medical devices, household appliances, or water purification.^14^ This makes AgNO_3_ a potential key component in the development of protective coatings for medical fabrics and air filtration systems. Even though there are studies regarding the antiviral activity of silver nanoparticles or chitosan alone, no scientific literature is present concerning the antiviral action of silver nitrate and bioactive glass as inorganic fillers in chitosan-based coatings.^7–9,16^ Moreover, the available studies do not consistently adhere to standardized ISO protocols for antibacterial and antiviral testing (ISO 21702 and ISO 22196), which limits the reproducibility and verification of the results. Considering that chitosan, BGs, and AgNO_3_ hold great potential to be incorporated as antifouling and bactericidal coating components on air, water, or industrial fluid filtration devices, the main goal of this research was to produce chitosan/BGs and chitosan/AgNO_3_ coatings on metallic filters by EPD. The coated filters were characterized in terms of their morphology, chemical composition, filtering capability, antibacterial properties against *S. aureus*, and antiviral activity against human coronavirus type OC43 (HCoV-OC43), human rhinovirus type A1 (HRV-A1), adenovirus type 5 (AdV-5), and influenza type A H3N2 strain (IFV-A H3N2).

## 2 Materials and Methods

### 2.1 Preparation of Chitosan/AgNO_3_ and Chitosan/BG Suspensions

A chitosan (molecular weight: 190–310 kDa, degree of deacetylation: 75–85%, Sigma-Aldrich, Taufkirchen, Germany) solution of 1 g/L was prepared in a solvent composed of distilled water and acetic acid (99%, Sigma-Aldrich) at a 20:1 volume ratio under magnetic stirring for 24 h. A 5 mM AgNO_3_ (≥99.5%, VWR, Darmstadt, Germany) solution was obtained by dissolving the AgNO_3_ salt in a previously prepared chitosan solution under stirring for 15 min. After complete dissolution, ethanol (99%, VWR) was added to the chitosan/AgNO_3_ solution to reach a final volume ratio of 79:1:20 (ethanol/acetic acid/water). The same preparation procedure was applied to obtain a 1 g/L BG suspension. The BG nanoparticles (mesoporous structure with nominal composition of 70 mol % SiO_2_ and 30 mol % CaO) were synthesized by using the microemulsion method as previously described,^17^ achieving a mean particle size of 120–150 nm.

### 2.2 Electrophoretic Deposition

The metallic filters were cleaned with an ethanol/acetone (1:1 vol %) mixture under ultrasonication, washed with deionized water, and air-dried prior to deposition. The EPD of chitosan/AgNO_3_ and chitosan/BG was performed by DC-EPD (Thurlby Thandar Instruments (TTi) EX752 M power supply, Huntingdon, UK) at room temperature by keeping the interelectrode distance at 10 mm and the applied voltage at 15 V. The deposition time for chitosan/AgNO_3_ and chitosan/BG was adjusted to 1 and 3 min, respectively. All of these parameters were chosen after several experiments based on a trial-and-error approach in order to obtain coatings with high surface homogeneity.

### 2.3 Scanning Electron Microscopy

The surface morphology of the coatings was analyzed by Field Emission Gun scanning electron microscopy (SEM) (FEG-SEM, Auriga CrossBeam, Carl Zeiss Microscopy GmbH, Jena, Germany). The metallic filters were placed on stubs fixed with conductive carbon tape, and the images were acquired at an accelerating voltage of 1 kV with a working distance in the range of 4–6 mm, depending on the magnification (90X-100 KX).

### 2.4 Energy-Dispersive X-ray Spectroscopy

An energy-dispersive X-ray spectrometer (EDX, X-MaxN Oxford Instruments, United Kingdom) coupled with SEM was used to generate elemental mappings and confirm the presence of the main coating components on the filters. EDX data were collected at an electron-accelerating voltage of 10 kV and a working distance of 6 mm.

### 2.5 Fourier Transform Infrared Spectroscopy

The chemical composition of all samples was analyzed by FTIR using a Shimadzu IRAffinity-1S instrument (Shimadzu Corp, Japan). The data were acquired in absorbance mode at the wavenumber in the range of 400 cm^–1^–4000 cm^–1^ at the resolution of 4 cm^–1^. For the apodization, a Happ-Genzel function was used.^18^

### 2.6 X-ray Diffraction

The phase composition of coatings was analyzed by XRD measurements using a Miniflex 600 HR (Rigaku, Japan) with Cu Kα radiation. The measurements were performed with a step size of 0.02° and speed of 4°/min over a 2θ range from 20° to 80°.

### 2.7 Bubble Point Test

The bubble point test is a nondestructive method of integrity testing, whose ultimate goal is to verify if the deposition of a coating on the filter leads to any changes in the filtration capability compared to the uncoated metallic filter. It returns the pressure value at which the surface tension of water inside the filter media is broken. This pressure level, known as the bubble point, is signaled by the first appearance of a steady stream of bubbles observed from a special filter holder through the downstream side of the filter media. The filter media bubble point is empirically measured by using specialized disc filter holders. These holders maintain a small observable liquid reservoir above and downstream of the membrane filter. Compressed air is applied against the upstream bottom surface of the membrane filter. The pressure is increased until a steady stream of bubbles emerges from the membrane through the water. The pressure at which the stream of bubbles occurs is called the bubble point. An illustration of the bubble-point test set up is provided in Figure 1.

**Figure 1:**
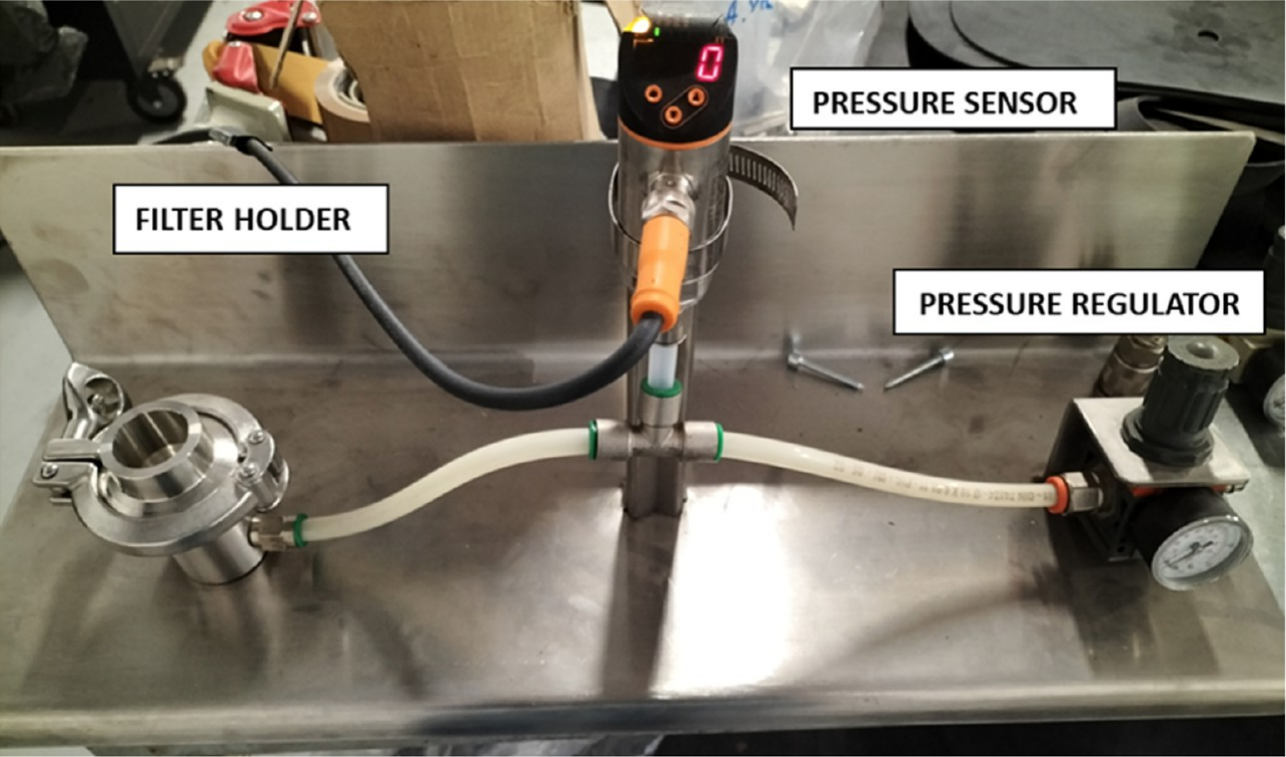
Bubble point test setup.

The self-made bubble point testing apparatus includes a relieving pressure regulator (Parker, P3XR Series, pressure range from 0 to 4000 mbar, temperature range from −40 to 60 °C, pressure rating of 16 bar.g), a pressure sensor (IFM, PN7099, pressure range from 1 to −1 mbar, temperature range from −25 to 80 °C), and a stainless-steel filter holder (GV FILTRI, GV-FH-SS-47, filter diameter of 47 mm, test filtration area of 10.7 cm^2^, pressure rating of 25 bar.g with nitrile butadiene rubber ensuring airtight sealing).

### 2.8 Antibacterial Test

The antibacterial performance of the new coatings was quantitatively evaluated in accordance with the ISO 22196 standard, a widely accepted protocol for assessing the antimicrobial activity of surfaces.^19^ Briefly, a suspension of *S. aureus* (ATCC 6538), a Gram-positive bacterial strain, was prepared and adjusted to a concentration of approximately 1 × 10^5^ colony-forming unit (CFU)/mL. An aliquot of 0.4 mL was inoculated onto the surface of uncoated control samples (uncoated metallic filters) as well as samples coated with chitosan/AgNO_3_ and chitosan/BG. A sterile polyethylene film was placed over each inoculum to ensure uniform spread across the surface. The inoculated samples were then incubated at 35 ± 2 °C for 20 h with 75% relative humidity. A schematic representation of the procedure is provided in Figure 2.

**Figure 2:**
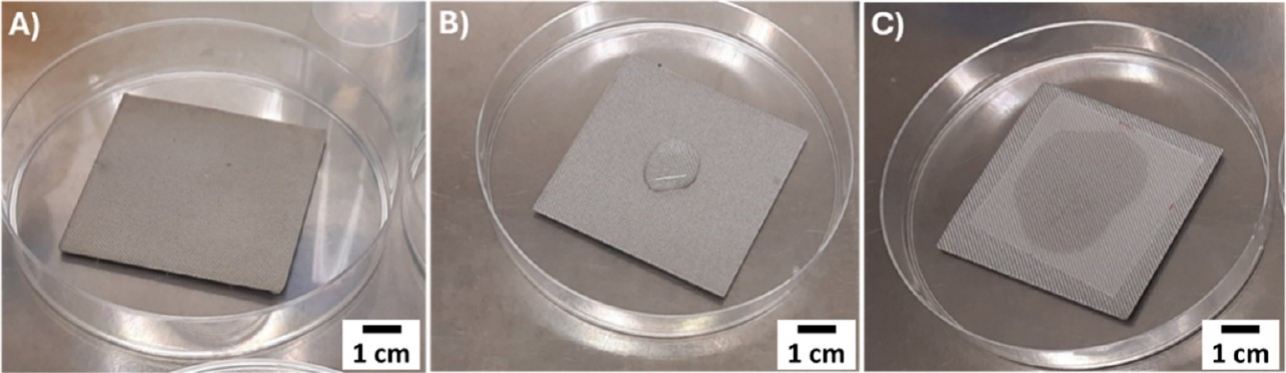
Methodology used to assess *S. aureus* viability on reference and coated samples, following ISO 22196 guidelines: (A) testing sample placed facing upward; (B) application of *S. aureus* inoculum onto the surface; (C) placement of parafilm over the inoculum to ensure uniform surface contact. Uncoated, chitosan/AgNO_3_- and chitosan/BG-coated samples were incubated for 20 h at 35 ± 2 °C.

Following incubation, the cover films were carefully removed, and each sample was washed with 10 mL of Soybean Casein Digest Lecithin Polysorbate (SCDLP) broth to recover the remaining viable bacteria. Serial dilutions (1:1, 1:10, 1:100; 1:1000) of the resulting suspensions were plated on Plate Count Agar and incubated at 35 ± 2 °C for 20 h. CFUs were enumerated to quantify the bacterial load on both coated and uncoated surfaces. Antibacterial activity was calculated by determining the *R* value (log reduction) and percentage inhibition, with the *R* value representing the logarithmic difference in bacterial counts between the untreated and treated surfaces. Prior to testing, both sample surfaces and cover films were sterilized via UV irradiation for 10 min in a laminar flow cabinet.

### 2.9 Cell Lines

Human lung fibroblasts (MRC-5) (ATCC CCL-171), human cervix adenocarcinoma cells (HeLa) (ATCC CCL-2), and Madine-Darby canine kidney epithelial cells (MDCK) (ATCC CCL-34) were cultured in Dulbecco’s Modified Eagle Medium (DMEM; Sigma-Aldrich) supplemented with heat-inactivated, 10% (v/v) fetal bovine serum (FBS; Sigma-Aldrich) in a humidified 5% CO_2_ incubator. Culture medium was supplemented with 1% (v/v) antibiotic-antimycotic solution (Zell Shield, Minerva Biolabs, Berlin, Germany).

### 2.10 Viruses

Human beta-coronavirus, type OC43 (HCoV-OC43, ATCC VR-1558) was produced on MRC-5 cells, and human rhinovirus, type A1 (HRV-A1, ATCC VR-1559) and adenovirus, type 5 (AdV-5, ATCC VR-5) were cultivated on HeLa cells, using DMEM, supplemented with heat-inactivated, 2% (v/v) FBS, in a humidified 5% CO_2_ incubator. Influenza virus type A (IFV-A H3N2, A/ChristChurch/28/03, Italian National Institute of Health) was produced on MDCK cells using DMEM, supplemented with 1 μg/mL of trypsin (Sigma-Aldrich), in a humidified 5% CO_2_ incubator.

Briefly, cells were seeded in tissue culture flasks at the optimal 80% subconfluency. Next, HCoV-OC43, HRV-A1, AdV-5, and IFV-A H3N2 were inoculated at multiplicity of infection 0.1 on the appropriate cell line, and flasks were incubated at 34 °C (HCoV-OC43, HRV-A1) or 37 °C (IFV-A H3N2, AdV-5). When a complete cytopathic effect occurred, supernatants and cells were harvested, pooled, frozen–thawed three times, then clarified, and aliquoted. IFV-A H3N2 and AdV-5 were concentrated by centrifugation (19,000g for 3h) before being aliquoted. Viral stocks were stored at −80 °C. Virus titration was performed by a standard focus assay (HCoV-OC43, HRV-A1, IFV-A H3N2) or standard plaque assay (AdV-5). Viral titers were expressed as focus-forming unit (FFU) per mL (HCoV-OC43, HRV-A1, IFV-A H3N2) or plaque-forming unit (PFU) per mL (AdV-5), as reported previously.^20^

### 2.11 Control Test for the Verification of the Cytotoxic Effect of Coatings on Host Cells

To determine the potential cytotoxic effect of chitosan/BG and chitosan/AgNO_3_ on host cells, experiments were performed according to the ISO 21702:2019­(E).^21^ In detail, four washes with 10 mL of soybean casein digest broth with lecithin and polyoxyethylene sorbitan monooleate (SCDLP broth) diluted 1:200 in DMEM 2% FBS (or DMEM in case of MDCK) were performed on metallic air filters coated with chitosan/BG or chitosan/AgNO_3_, and on the uncoated material. 10 mL of diluted broth that did not contact any material was used as the negative control. Cells were seeded in 96-well plates and treated with pure or 1:10 diluted wash-out solutions for 1 h at 34 °C (MRC-5, HeLa for HRV-A1), 1 h at 37 °C (MDCK) or 2 h at 37 °C (HeLa for AdV-5). After a wash with culture medium, DMEM 2% FBS (MRC-5, HeLa for HRV-A1), DMEM (MDCK), or Sea Plaque (HeLa for AdV-5) was added to cells, which were then incubated for 16 or 24 h at 34 °C (MRC-5, HeLa for HRV-A1),16 or 96 h at 37 °C (MDCK, HeLa for AdV-5). Cell viability was observed with an optical microscope in order to evaluate the alterations of treated cell monolayers.

### 2.12 Control Test for the Verification of Cell Sensitivity to Virus and the Inactivation of Antiviral Activity by SCDLP Broth

To verify the cell sensitivity to virus and the inactivation of antiviral activity, experiments were performed according to the ISO 21702:2019­(E).^21^ Four washes with 10 mL SCDLP broth diluted 1:200 in DMEM 2% FBS (or DMEM in case of IFV-A H3N2) were performed on the materials with chitosan/BG or chitosan/AgNO_3_ coatings and on the uncoated material. 10 mL of diluted broth that did not contact any material was used as the negative control. 50 μL of pure virus was added to 5 mL of each wash-out solution. Test and control conditions were incubated for 30 min at 25 °C. Next, virus suspensions were titrated, and viral titers were calculated by the mean of 8 technical replicates.

### 2.13 Antiviral Assays

To determine the antiviral potential of chitosan/BG and chitosan/AgNO_3_ coatings, antiviral assays were performed according to the ISO 21702:2019­(E) standard guidelines.^21^ In detail, 400 μL of the viral stock (titer of 10^8^ FFU or PFU/mL) was deposited on a 5 × 5 cm square of material with chitosan/BG or chitosan/AgNO_3_ and on the uncoated material. The viral suspension was covered by a stomacher bag (cut in a square of 4 × 4 cm), creating a thin film to maximize the contact with the material and to avoid virus evaporation. Four washes with 10 mL of SCDLP broth diluted 1:200 in DMEM 2% FBS (or DMEM in case of IFV-A H3N2) were performed to collect the virus at different time-points: immediately (only for the uncoated material) or after 24 h at 25 °C (for coated and uncoated material) in a humidified 5% CO_2_ incubator (humidity >90%). Next, virus suspensions were titrated, and viral titers per mL (FFU/mL or PFU/mL) and viral titers per cm^2^ of surface (FFU/cm^2^ or PFU/cm^2^) were calculated by means of 8 technical replicates.

Viral titer per cm^2^ of the surface (FFU/cm^2^ or PFU/cm^2^) was calculated using the following formula

$$\frac{viral\;titer\;({FFU/cm}^{2}\text{or}\;\;{PFU/cm}^{2})=[viral\;titer(FFU/mL\;\text{or}\;PFU/mL)\times V\,\;\;(volume\;of\;diluted\;SCDLP\;broth\;added\;to\;the\;\;specimen,in\;mL)]}{A(surface\;area\,\;\;of\;the\;cover\;film,\;in{\;cm}^{2})}$$

Antiviral activity was expressed as *R* value [(log viral titer (FFU or PFU/cm^2^) collected from the coated material after 24 h at 25 °C)—(log viral titer (FFU or PFU/cm^2^) immediately collected from the uncoated material)]—[(log viral titer (FFU or PFU/cm^2^) collected from the uncoated material after 24 h at 25 °C)—(log viral titer (FFU or PFU/cm^2^) immediately collected from the uncoated material)] and inhibition percentage 100—[(viral titer (FFU or PFU/cm^2^) collected from the coated material after 24 h at 25 °C)/(viral titer (FFU or PFU/cm^2^) collected from the uncoated material after 24 h at 25 °C *100].

### 2.14 Drying Test

To assess the direct action of coatings on viral particles, 75 μL of virus with a titer of 10^8^ FFU/mL was deposited onto a 2.5 × 2.5 cm square of the coated and uncoated materials and left under a biological hood for 3 h at room temperature, until the viral inoculum was completely dried. Four washes with 500 μL of DMEM and 2% FBS were performed to recover the virus from the coated and uncoated materials. Next, the wash-out solutions were titrated, and viral titers were calculated as previously described. The antiviral activity is expressed in *R* and % of inhibition.

### 2.15 Ion Release Test

To conduct the ion release test, 100 μL of DMEM 2% FBS was deposited on a 2.5 × 2.5 cm square of the coated or uncoated material and covered by a stomacher bag (cut in a square of 2 × 2 cm). After 3 or 24 h incubation at 25 °C in a humidified 5% CO_2_ incubator (humidity >90%), four washes with 800 μL of DMEM 2% FBS were performed and 100 μL of pure virus was added to 300 μL of each wash-out solution. The wash-out solutions with viral inoculum were incubated for 3 or 24 h at 25 °C in a humidified 5% CO_2_ incubator (humidity >90%). Next, virus suspensions were titrated, and viral titers were calculated as previously described. The antiviral activity is expressed in *R* and % of inhibition.

## 3 Results and Discussion

### 3.1 Surface Morphology

The surface morphology of chitosan/AgNO_3_ and chitosan/BG coatings was examined by SEM (Figure 3). The images confirm the deposition of continuous, well-adherent, and crack-free chitosan-based coatings. High-magnification images show clusters of BGs and AgNO_3_, forming some agglomerates. Similar morphological features of chitosan/BG and chitosan/Ag coatings were observed by Pishbin et al. previously.^22^ However, no blockage of the filter pores can be seen for both coating types, which indicates the suitability of the used coating materials and deposition technique for the intended application of air and water filtration.

**Figure 3:**
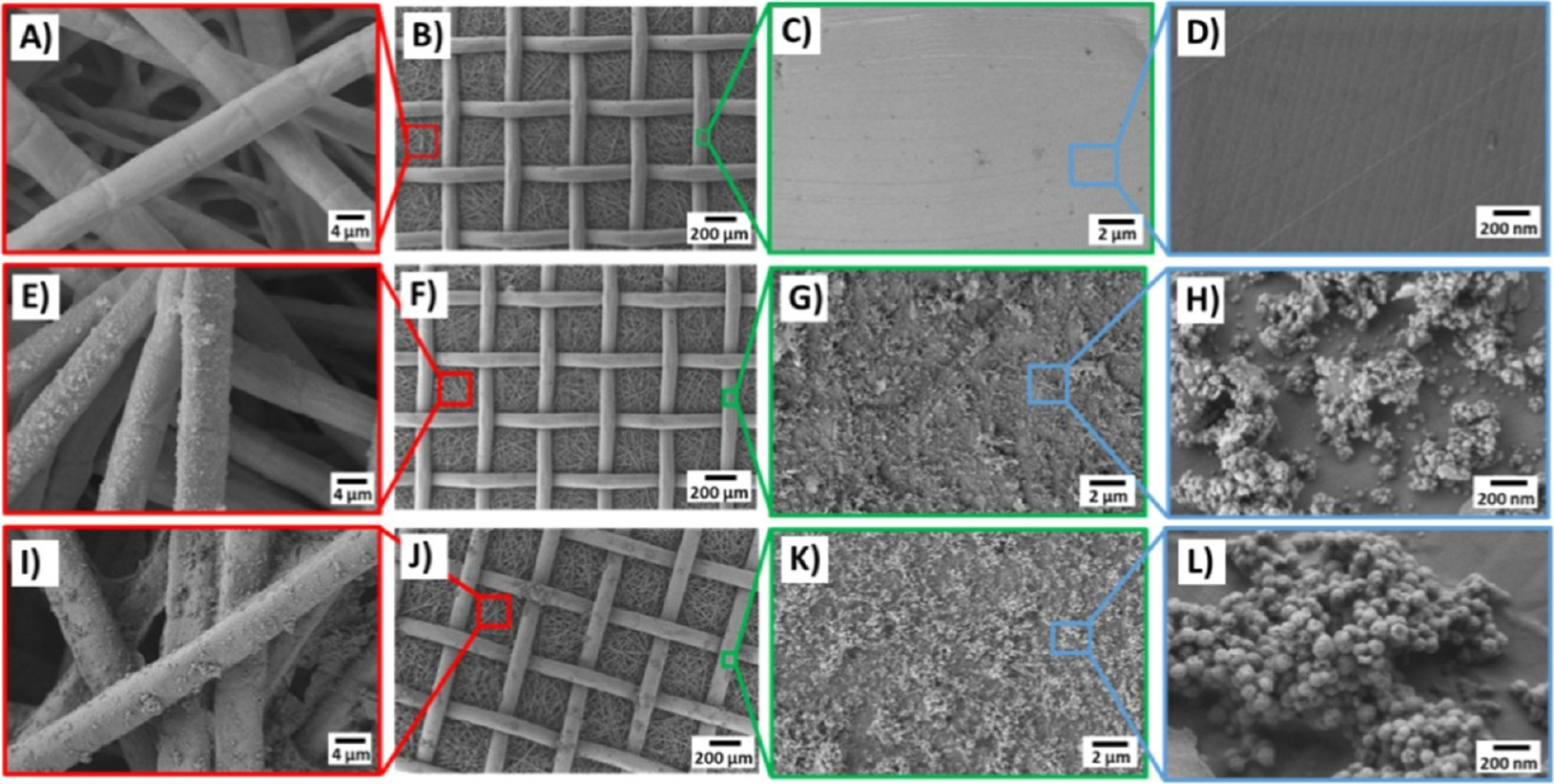
SEM micrographs of (A–D) uncoated, (E–H) chitosan/AgNO_3_ -coated and (I–L) chitosan/BG-coated metallic filters at different magnifications.

### 3.2 Chemical Composition

The chemical composition of chitosan/AgNO_3_ and chitosan/BG coatings was examined by EDX and FTIR analyses (Figures 4 and 5). According to the EDX analysis, peaks assigned to Ni, Cr, Mn, Fe, and Mo appear in the spectra of both chitosan/AgNO_3_- and chitosan/BG-coated filters, which are related to the chemical composition of the metallic substrate. Next to the presence of C and O peaks associated with the chitosan structure, the presence of Ag is confirmed in the spectrum of chitosan/AgNO_3_ coatings. In the case of chitosan/BG coatings, the successful deposition of the inorganic filler is verified by the peaks related to Si and Ca.

**Figure 4:**
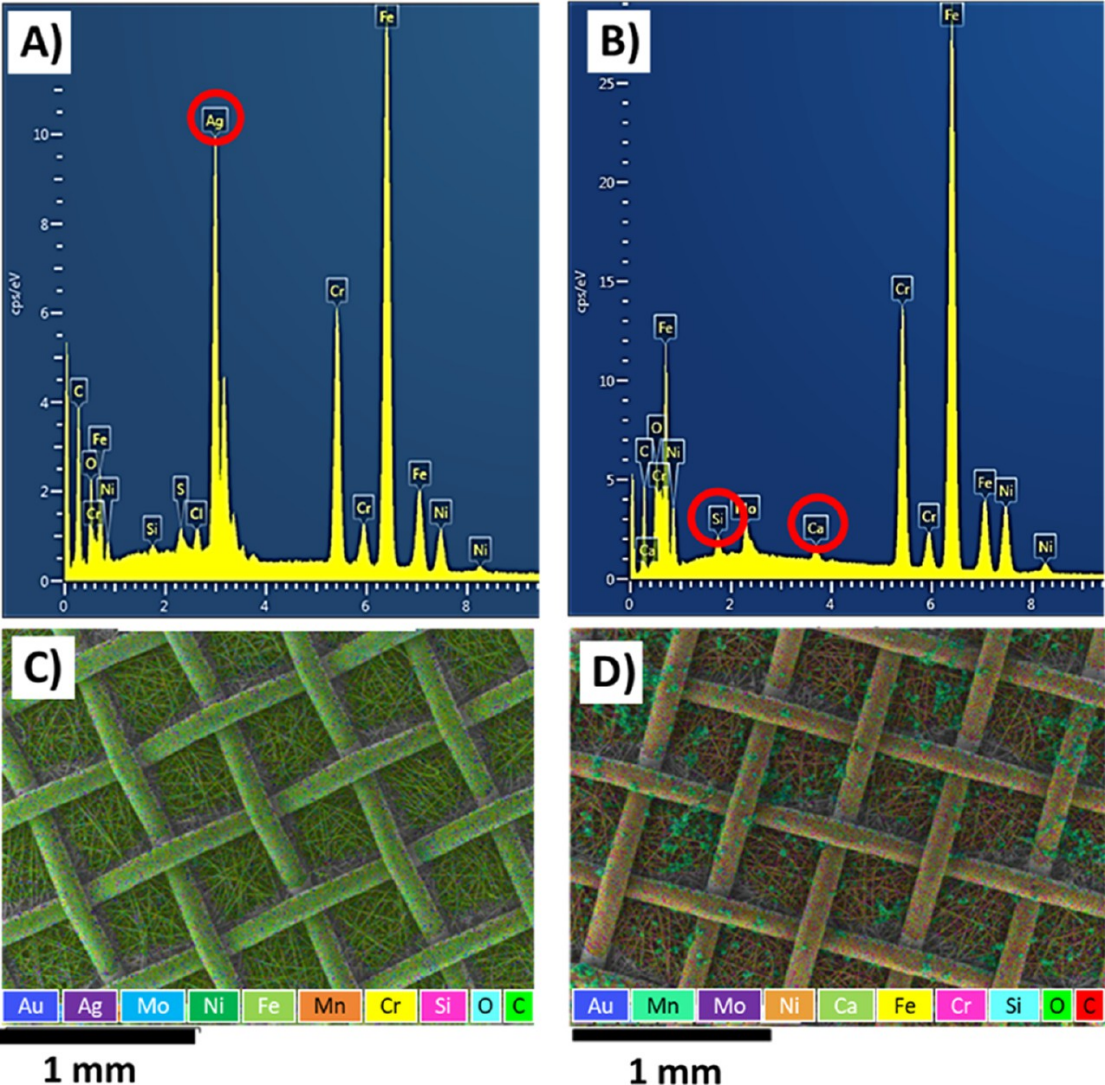
EDX analysis and elemental mapping of (A,C) chitosan/AgNO_3_-coated and (B,D) chitosan/BG-coated filters.

**Figure 5:**
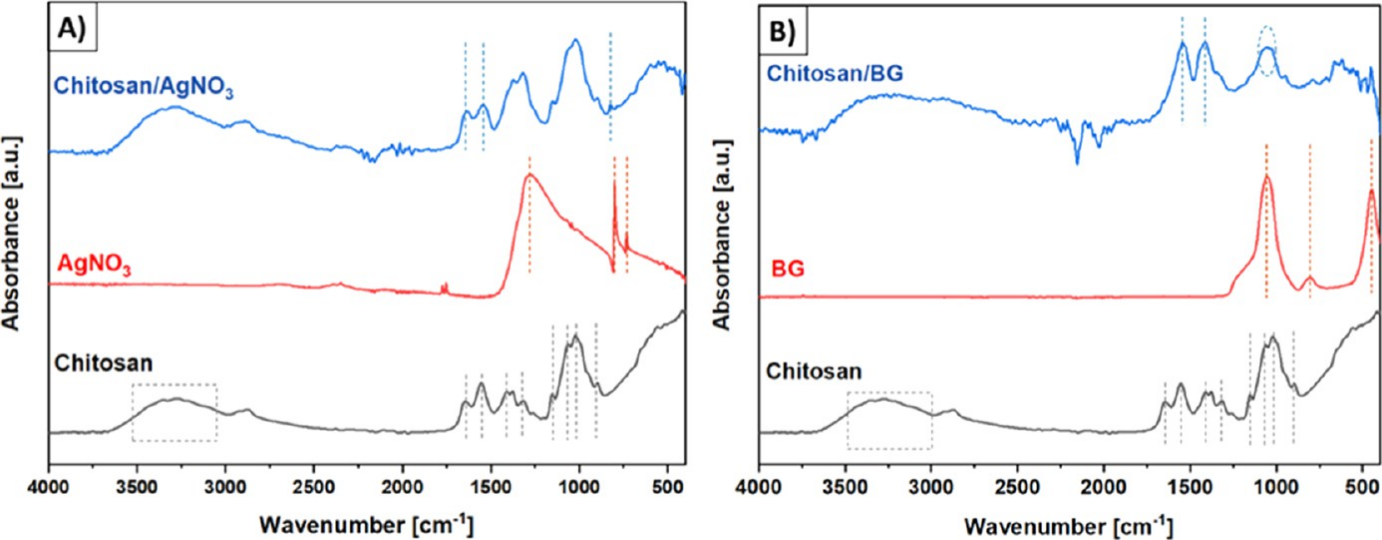
FTIR spectra of (A) chitosan/AgNO_3_-coated and (B) chitosan/BG-coated filters. The relevant FTIR peaks are discussed in Section 3.2.

The FTIR spectrum of pure chitosan powder exhibits a broad band in the range of 3400–3200 cm^–1^, which is related to the overlapping of the stretching vibration of –NH_2_ and the asymmetrical stretching vibration of –OH.^23^ Additionally, the peaks at 2875 cm^–1^, 1650 cm^–1^, 1550 cm^–1^, and 1410 cm^–1^ represent the –CH_2_ asymmetric stretching vibrations, the C=O stretching of the amide I band, the –NH bending vibrations of the amide II band, and the C–H deformation, respectively.^24,25^ The bands at 1375 cm^–1^ and 1310 cm^–1^ can be assigned to the CH_3_ symmetrical deformation mode and the C–N stretching vibration in the amide III band, respectively.^26,27^ Furthermore, the band in the range of 1150–1000 cm^–1^ and the peak 895 cm^–1^ are associated with C–O–C bands and the CH deformation of the β-glycosidic bond.^27^ The FTIR spectrum of AgNO_3_ is characterized by a main peak at 1300 cm^–1^, which is related to the vibration of the NO_3_.^28^ The peaks in the range of 700 cm^–1^ to 800 cm^–1^ are assigned to the nitro compounds in pure AgNO_3_.^29^ In the case of chitosan/AgNO_3_ coatings, the main absorbance peaks of chitosan are preserved but they shift to lower wavenumbers. In particular, the peak at 1650 cm^–1^ shifts to 1630 cm^–1^, which could be indicative of the coordination bond formation between the silver atom and the oxygen/nitrogen in chitosan.^25^ Moreover, a new peak at 825 cm^–1^ appears, which is related to the stretching vibration of the chitosan/Ag complex.^25^

The FTIR spectrum of pure BGs exhibits peaks at 450 cm^–1^, 800 cm^–1^, and 1050 cm^–1^, which are assigned to the Si–O–Si rocking mode and symmetric stretching mode, respectively.^17,30^ In the case of chitosan/BG coatings, most of the peaks in the range between 1400 cm^–1^ and 1200 cm^–1^, previously observed in the chitosan spectrum, disappear. Additionally, the double peak at 1050 cm^–1^ characteristic of the glycosidic bond in chitosan merges into a single peak at the same position after the addition of BG in the coating. Furthermore, the broadening of the O–H and N–H stretching bands (around 3200–3500 cm^–1^) suggests the formation of hydrogen bonds between the hydroxyl (–OH) groups of BG and the amino (–NH_2_) or hydroxyl (–OH) groups of chitosan.^31^ In addition, the shifts of the peaks at 1560–1650 cm^–1^ indicate changes in the NH_3_^+^ or COO^–^ bending vibrations and thus hydrogen bonding or electrostatic interaction between the amine or carboxyl in chitosan and the silanol groups of BG.^32^

The XRD patterns of uncoated, chitosan/AgNO3, and chitosan/BG-coated filters are shown in Figure 6. The peak at 2θ 75°, which can be observed for uncoated, chitosan/AgNO_3_-and chitosan/BG-coated filters, is associated with the austenite phase γ-(220) of stainless steel.^33,34^ Chitosan/BG coatings exhibit an amorphous structure, considering the lack of any strong characteristic reflection peaks in their pattern. In contrast, the peak at 2θ 38° in the XRD pattern of chitosan/AgNO_3_ coatings is related to the cubic phase (111) of Ag and confirms the crystalline nature of the coatings.^35,36^

**Figure 6:**
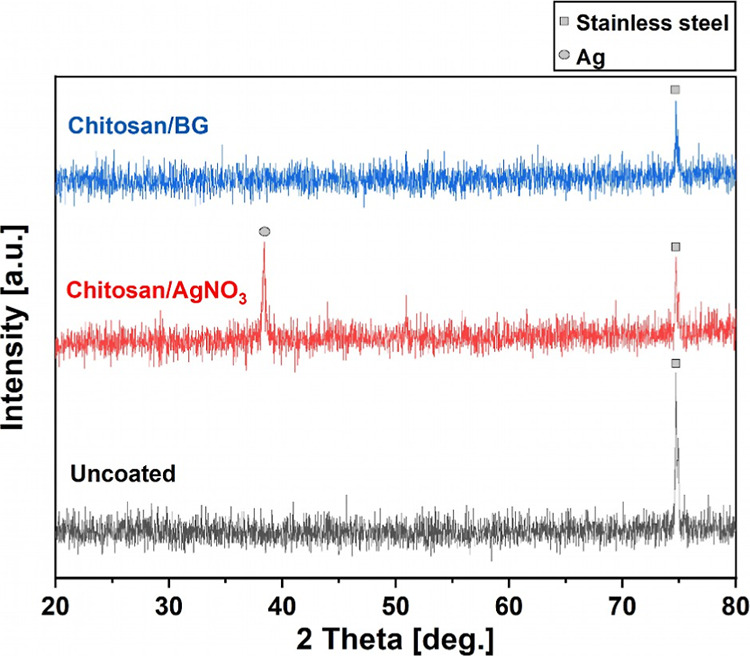
XRD patterns of uncoated, chitosan/AgNO_3_, and chitosan/BG-coated filters. The relevant peaks are discussed in Section 3.2.

During EPD, Ag ions present in the AgNO_3_-containing suspensions are reduced to Ag^0^ due to the presence of electrons at the cathode.^22^ Furthermore, the production of OH^–^, resulting from the water hydrolysis during EPD, contributes to the cathodic reduction of Ag.^37^ As a result, metallic crystalline Ag is deposited within the chitosan coating matrix in the presence of an electric field. It should be noted that AgNO_3_ is light-sensitive; thus, in addition to the electrochemical reduction during EPD, photochemical reactions in the presence of visible light could also contribute to Ag reduction.^38^

### 3.3 Filtration Capability

A bubble point test (*n* = 3) was performed in order to evaluate the filtration capability of the filters after applying chitosan/BG and chitosan/AgNO_3_ coatings (Table 1). The results showed that the bubble point values for both chitosan/BG- and chitosan/AgNO_3_-coated filters are similar to those for the uncoated filter. Chitosan/BG coatings exhibited the highest bubble point values among all tested samples. It should be noted that there is no precise threshold value that indicates a suitable filtration capability but generally, a higher bubble point value is characteristic for a greater blockage of the filter. However, the measured increase in the bubble point value after the deposition of chitosan/BG and chitosan/AgNO_3_ coatings is minimal and does not indicate significant changes in the filtering capacity of the filter media. In particular, the observed differences in the bubble point values of uncoated and coated filters are minor (≤15 mbar) compared to the overall filtration pressure drop, and can be considered negligible compared to the typical operating pressure ranges of filtration devices for the targeted applications (e.g., in water filters^39^). Thus, considering the practical rather than purely statistical significance of the results, we can conclude that the coating deposition does not affect the functional performance of the filters in practical use.

**Table 1:** Bubble Point Values [mbar] of Uncoated Metallic Filters, Chitosan/BG-Coated Filters, and Chitosan/AgNO_3_-Coated Filters

sample	bubble point value [mbar]
uncoated metallic filter	128 ± 2
chitosan/BG-coated filter	143 ± 2
chitosan/AgNO_3_-coated filter	137 ± 2

For industrial applications, it is essential to ensure that the coating does not influence the filtration processes and keeps the filter pores open in order to avoid pressure drops with consequently higher pumping power, energy consumption, and increased costs. It should be noted that the key point of the coatings is not increasing the filtration effect but keeping the filter clean, as potential bacterial accumulation (and growth) can eventually block the filter, leading to the need to substitute or clean the filter. Moreover, it has been reported previously that the bacteria grown under certain conditions could be released from the filter, which could pose additional health risks.^40^

### 3.4 Antibacterial Assay

The antibacterial efficacy of the chitosan/AgNO_3_- and chitosan/BG-coated samples was assessed by quantifying the number of viable *S. aureus* bacteria recovered from both coated and uncoated reference surfaces. The corresponding results are presented in Figure 7 and summarized in Table 2. While a substantial number of viable bacteria were recovered from the uncoated samples, a marked reduction in bacterial counts was observed on both coated surfaces. This indicates the effective antibacterial action of the coatings. Specifically, the chitosan/AgNO_3_ and chitosan/BG coatings exhibited antibacterial efficiencies exceeding 99%, as evidenced by the minimal recovery of viable *S. aureus* compared to the control, thereby confirming their potent antimicrobial activity. The antibacterial effect observed in the chitosan/AgNO_3_-coated filters against *S. aureus* can be primarily attributed to the well-established antimicrobial properties of Ag incorporated within the coating matrix. Silver ions (Ag^+^) exert broad-spectrum antibacterial activity through multiple mechanisms, including disruption of bacterial cell membrane integrity, interference with intracellular processes such as DNA replication, and induction of reactive oxygen species, which lead to oxidative stress and irreversible cellular damage. These synergistic mechanisms contribute to the potent bactericidal activity of silver-based coatings.^41–43^ In contrast, uncoated samples lack intrinsic antimicrobial properties and thus allow for bacterial viability and proliferation. These findings are consistent with previous reports demonstrating the potent antibacterial efficacy of silver-containing coatings against *S. aureus*, further validating the effectiveness of chitosan-based Ag delivery systems.^44–46^ In parallel, the functionalization of chitosan with BG also demonstrated effective antibacterial activity against *S. aureus*, as evidenced by the negligible number of viable bacteria recovered following exposure to the chitosan/BG-coated samples (Figure 7C). The antibacterial effect observed is in line with previous studies that have reported the inhibitory activity of bioglass-based materials against *S. aureus*.^47–49^ It is also important to emphasize that the observed antibacterial activity of the coated filters may, in part, be attributed to chitosan itself, a biopolymer widely recognized for its intrinsic antimicrobial properties. The antibacterial action of chitosan is primarily associated with its polycationic nature, which enables strong electrostatic interactions with the charged bacterial cell membranes. These interactions can compromise membrane integrity, increase permeability, and lead to the leakage of intracellular constituents. Furthermore, chitosan has the capacity to chelate essential nutrients and metal ions, disrupt microbial metabolic pathways, penetrate bacterial cells to interfere with nucleic acid synthesis, lead to damaged bacterial organelles, hinder biochemical processes, and cell death.^6^ These multifaceted mechanisms collectively contribute to its broad-spectrum efficacy against *S. aureus* and other pathogenic microorganisms.^50^ Chitosan has been previously incorporated into coatings for medical implants to reduce biofilm formation,^51^ into wound dressings to promote healing and prevent infection,^52^ and into antimicrobial films and hydrogels that show enhanced bactericidal performance.^53,54^

**Figure 7:**
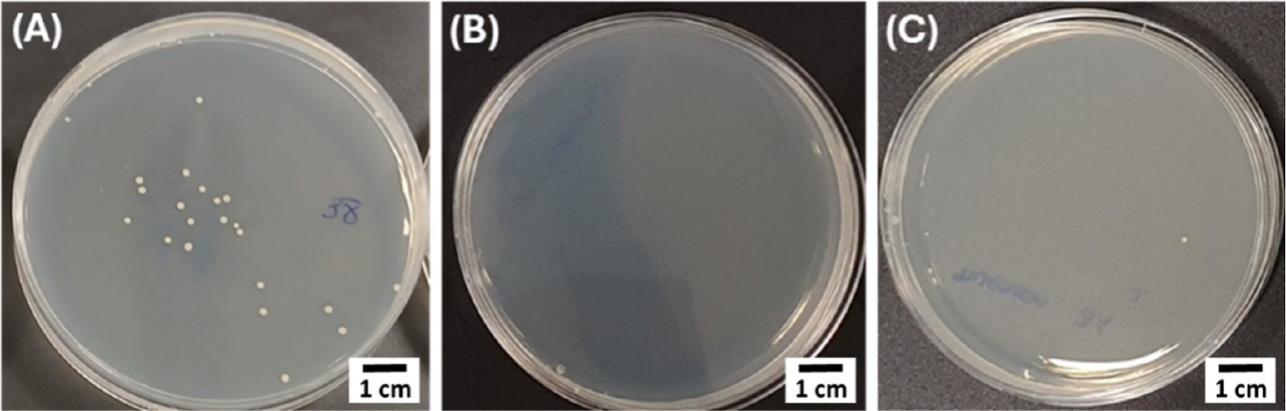
Antibacterial test representative CFUs results for the (A) uncoated sample (1:100 dilution), (B) chitosan/AgNO_3_ coating (1:1 dilution), and (C) chitosan/BG coating (1:1 dilution).

**Table 2:** Results of the Antibacterial Activity of Chitosan/AgNO_3_ and Chitosan/BG Coatings against *S. aureus* Bacteria Expressed in *R* Value and Inhibition %

sample	CFU/cm^2^ after 24 h incubation	*R* value	inhibition %
uncoated	2.09 × 10^4^	3.5	99.97
chitosan/AgNO_3_	0.0 × 10°		
uncoated	2.09 × 10^4^	3.28	99.94
chitosan/BG	1.0 × 10^0^		

### 3.5 Antiviral Assays

The potential antiviral activity of chitosan/AgNO_3_ and chitosan/BG coatings was assessed following the ISO guidelines (ISO 21702:2019­(E)^21^). The antiviral action of the coatings was investigated against four different respiratory viruses: three RNA viruses, HCoV-OC43, IFV-A H3N2, and HRV-A1, and one DNA virus, AdV-5. The antiviral tests were preceded by control tests according to the ISO standards.

The first control test regards the assessment of any cytotoxic effect of the coatings on host cells by evaluating potential alterations of the treated cell monolayers. Coated and uncoated filters (used as a control sample) were washed with SCDLP broth, a typical neutralizer used to inactivate antimicrobial and antiviral agents, diluted in culture medium. Wash-out solutions were incubated with the different cell types with the same experimental conditions as the antiviral tests, and cytotoxicity was assessed by observation under an inverted microscope. Pure and 1:10 diluted wash-out solutions of chitosan/AgNO_3,_ chitosan/BG coatings or uncoated sample were incubated for 16 h on MRC-5 cells (for HCoV-OC43), for 24 h (for HRV-A1) or 96 h (for AdV-5) on Hela cells and for 16 h on MDCK cells (for IFV-A H3N2). As shown in Figure 8, pure and diluted wash-out solutions did not exhibit any toxic effect on all tested cell lines, demonstrating good cytocompatibility of the studied coatings.

**Figure 8:**
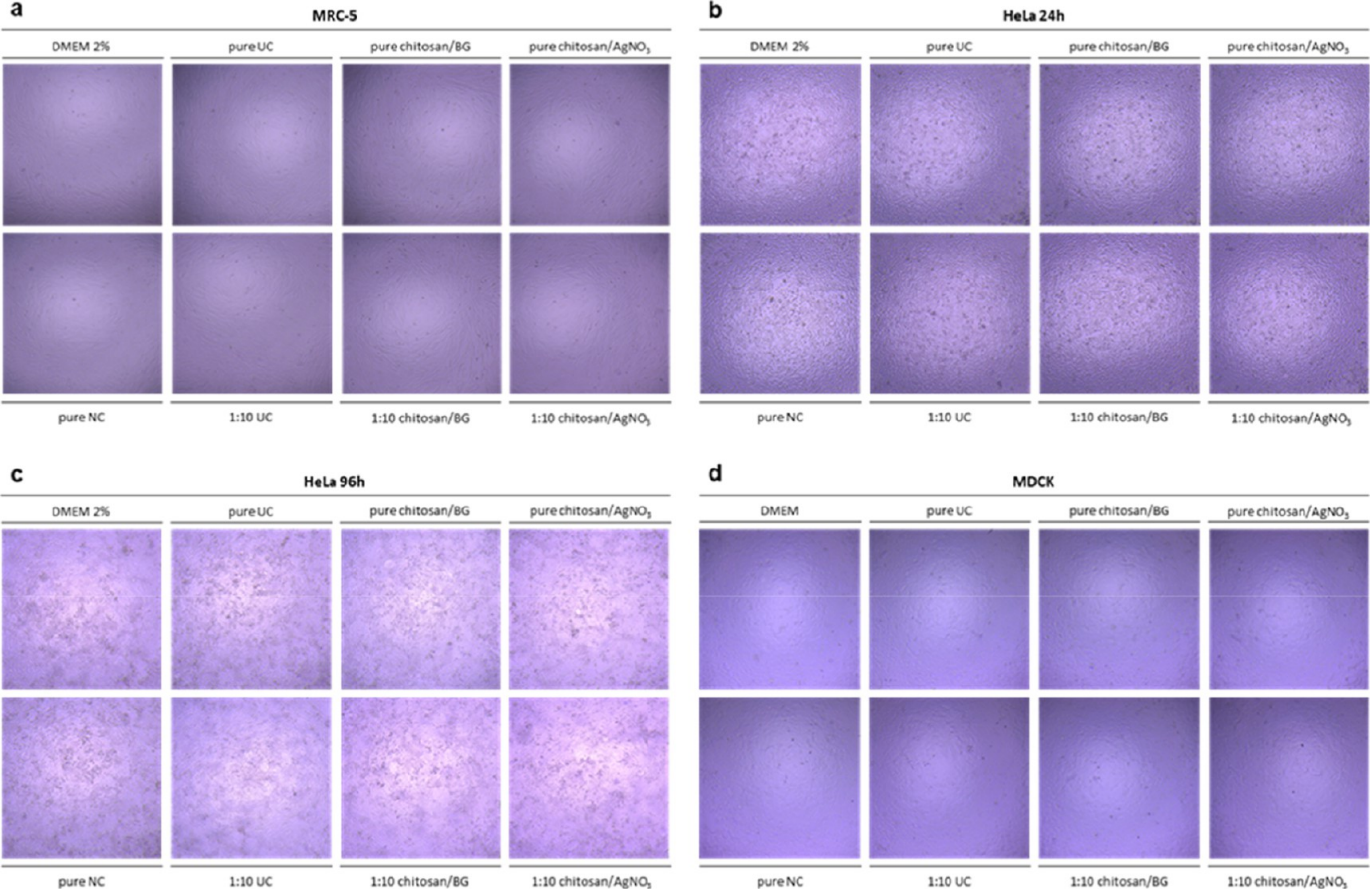
Metallic air filters coated with chitosan/BG or chitosan/AgNO_3_ showed no cytotoxicity on any tested cell lines. Cytotoxicity assessment was performed on (a) MRC-5 for HCoV-OC43, (b) HeLa 24 h for HRV-A1, (c) HeLa 96 h for AdV-5, and (d) MDCK for IFV-A H3N2. The cytotoxic effect of coatings on host cells was analyzed via optical microscope observation as reported in ISO guideline (a–d). Magnification, 400×. DMEM, culture medium; DMEM 2%, culture medium supplemented with 2% of serum; pure, pure wash-out solution; 1:10, 1:10 diluted wash-out solution.

The second control test considers the verification of the cell sensitivity to the virus and the inactivation of antiviral activity by SCDLP broth. As reported in Table 3 and Figure 9, the logarithmic difference between viral titers of wash-out solutions from chitosan/AgNO_3_, chitosan/BG coatings or the uncoated sample, and that from NC resulted lower than 0.5 for all the tested viruses, satisfying the condition for the verification of the test.

**Table 3:** Numerical Results of the Second Control Test Required by the ISO 21702:2019­(E) Guidelines^a^ ^,^ ^21^

virus	sample	titer (FFU/mL or PFU/mL)	log (titer)
HCoV-OC43	NC	1.50 × 10^6^	6.18
	UC	2.27 × 10^6^	6.36
	chitosan/BG	1.79 × 10^6^	6.25
	chitosan/AgNO_3_	1.49 × 10^6^	6.17
HRV-A1	NC	3.99 × 10^5^	5.60
	UC	4.51 × 10^5^	5.65
	chitosan/BG	4.23 × 10^5^	5.63
	chitosan/AgNO_3_	4.44 × 10^5^	5.65
AdV-5	NC	1.06 × 10^6^	6.02
	UC	1.45 × 10^6^	6.16
	chitosan/BG	2.29 × 10^6^	6.36
	chitosan/AgNO_3_	8.94 × 10^5^	5.95
IFV-A H3N2	NC	3.21 × 10^6^	6.51
	UC	2.62 × 10^6^	6.42
	chitosan/BG	3.00 × 10^6^	6.48
	chitosan/AgNO_3_	2.94 × 10^6^	6.47

a NC: negative control; UC: uncoated.

**Figure 9:**
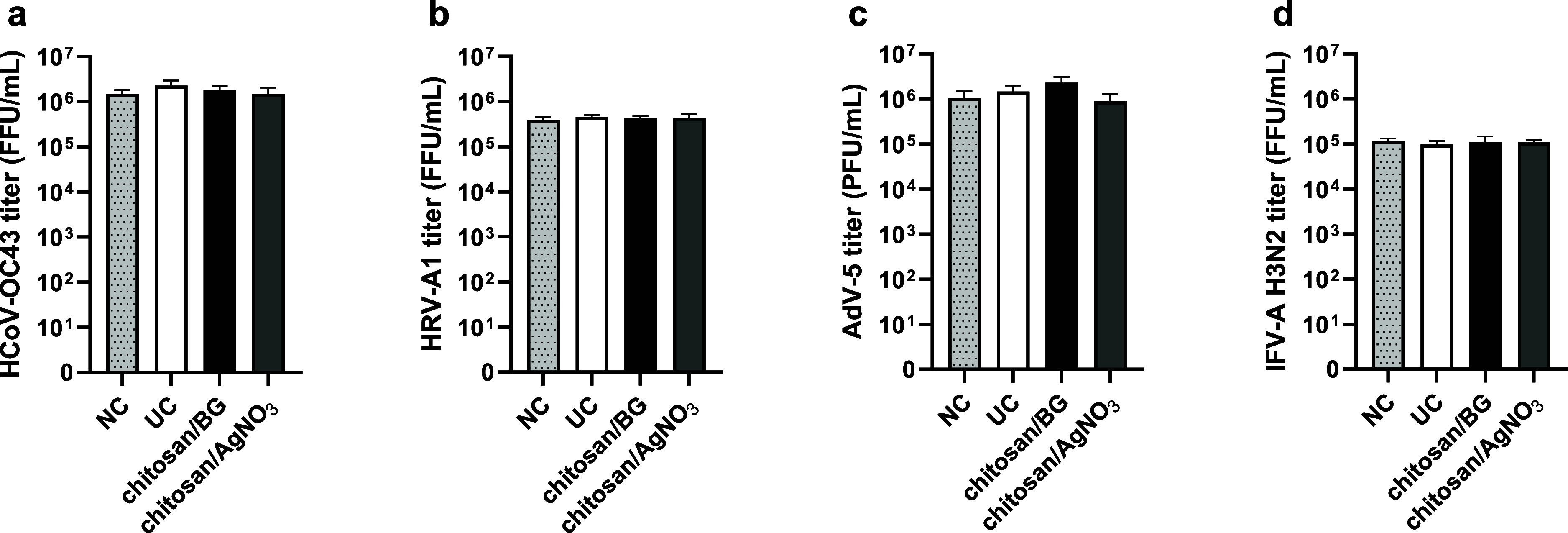
SCDLP broth inactivated the potential antiviral activity of the coated metallic air filters, and cell sensitivity to virus infection was not modified. Control tests were conducted against (a) HCoV-OC43, (b) HRV-A1, (c) AdV-5, (d) IFV-A H3N2. Viral titer retrieved by wash-out solutions after 30 min at 25 °C of incubation is reported on the *Y*-axis and expressed as FFU or PFU per mL (FFU or PFU/mL). Sample type is reported on *X*-axis. NC, negative control; UC, uncoated. Additional details of the assay protocol are reported in the Materials and Methods section.

Since both control conditions were verified for all viruses, the antiviral activity of the coatings was investigated, according to the antiviral test described in the ISO 21702:2019­(E).^21^

As reported in Table 4, a slight reduction of 1 logarithm of viral titer recovered after incubation with the uncoated material compared to the non-incubated virus (UC Imm) was reported in the case of HRV-A1, AdV-5, and IFV-A H3N2. This decrease after incubation might be ascribed to susceptibility of the viruses to incubation at 25° for 24 h.

**Table 4:** Numerical Results of the Antiviral Assays Conducted Following the ISO21702:2019­(E) Guidelines^a^ ^,^ ^21^

virus	sample	titer (FFU/mL or PFU/mL)	titer (FFU/cm^2^ or PFU/cm^2^)	*R* value	inhibition %
HCoV-OC43	UC Imm.	2.81 × 10^6^	1.75 × 10^6^	-	-
	UC	8.25 × 10^1^	5.16 × 10^1^	-	-
	chitosan/BG	1.49 × 10^2^	9.30 × 10^1^	–0.26	–14.95
	chitosan/AgNO_3_	2.00 × 10^1^	1.25 × 10^1^	0.62	35.94
HRV-A1	UC Imm.	2.06 × 10^6^	1.29 × 10^6^	-	-
	UC	4.46 × 10^5^	2.79 × 10^5^	-	-
	chitosan/BG	6.26 × 10^1^	3.91 × 10^1^	3.85	99.99
	chitosan/AgNO_3_	1.26 × 10^1^	1.14 × 10^1^	4.39	99.99
AdV-5	UC Imm.	5.24 × 10^6^	3.28 × 10^6^	-	-
	UC	6.99 × 10^5^	4.36 × 10^5^	-	-
	chitosan/BG	1.49 × 10^1^	1.21 × 10^1^	4.56	99.99
	chitosan/AgNO_3_	4.05 × 10^5^	2.53 × 10^5^	0.24	41.86
IFV-A H3N2	UC Imm.	2.90 × 10^5^	1.81 × 10^5^	-	-
	UC	1.15 × 10^4^	7.17 × 10^3^	-	-
	chitosan/BG	1.79 × 10^3^	1.12 × 10^3^	0.81	84.36
	chitosan/AgNO_3_	2.00 × 10^2^	1.24 × 10^2^	1.76	98.26
					

a UC Imm.: uncoated immediately collected; UC: uncoated.

Surprisingly, a reduction of 4 logarithms was evidenced comparing the viral titer of HCoV-OC43 recovered after incubation on the uncoated sample and the non-incubated virus (UC Imm). To differentiate if the decrease of HCoV-OC43 titer was due to a time-dependent low persistence of the virus at 25 °C or to an inhibitory activity exerted by the uncoated material, the antiviral test was repeated incubating 400 μL of high titer virus onto a sterile plastic Petri dish at 25 °C for 24 h, as an additional control condition (no sample), even if not included in the ISO guidelines. Results of the antiviral test with the additional control condition are reported in Table 5. In this case, only a slight reduction of 1 logarithm of HCoV-OC43 titer recovered after incubation on Petri dish (no sample) compared to the non-incubated virus (UC Imm) was reported, in line with the reduction of viral titer after incubation with the uncoated material observed for the other tested viruses. These data allowed us to conclude that the uncoated air filter exerts an intrinsic antiviral activity against HCoV-OC43.

**Table 5:** Numerical Results of the Antiviral Assay against HCoV-OC43 with Additional NO SAMPLE Control^a^

virus	sample	titer (FFU/mL or PFU/mL)	titer (FFU/cm^2^ or PFU/cm^2^)	*R* value	inhibition %
HCoV-OC43	UC Imm.	1.62 × 10^6^	6.44 × 10^5^	-	-
	NO SAMPLE	3.53 × 10^5^	2.21 × 10^5^	-	-
	UC	3.59 × 10^1^	7.79 × 10°	4.45	99.99
	chitosan/BG	5.86 × 10^2^	4.71 × 10^2^	2.67	99.79
	chitosan/AgNO_3_	1.04 × 10^1^	3.52 × 10°	4.80	99.99

a UC Imm.: uncoated immediately collected; UC: uncoated.

Results of the antiviral tests of chitosan/AgNO_3_ and chitosan/BG coatings are reported in Table 6 and are depicted in Figure 10. *R* values and percentage of inhibition were calculated as reported in the Materials and Methods section. The two coatings exerted a strong antiviral activity against HRV-A1, displaying a nearly complete inactivation of the virus, with an *R* value of 4.39 for the chitosan/AgNO_3_ coating (inhibition %: 99.99) and an *R* value of 3.85 for the chitosan/BG coating (inhibition %: 99.99). A significant antiviral activity was reported also for chitosan/BG against AdV-5, with an *R* value of 4.56 (inhibition %: 99.99), while a mild action against AdV-5 was exhibited for chitosan/AgNO_3_, with an *R* value of 0.24 (inhibition %: 41.86). A significant inhibition of IFV-A H3N2 infectivity was observed with an *R* value of 1.76 for chitosan/AgNO_3_ (inhibition %: 98.26) and 0.81 for chitosan/BG (inhibition %: 84.36).

**Table 6:** Summary of the Antiviral Activity of Chitosan/BG and Chitosan/AgNO_3_ Coatings against Respiratory Viruses Expressed in *R* Value and Inhibition %^a^

coating	virus	*R* value	inhibition %
chitosan/BG	HCoV-OC43	–0.26 (2.67*)	–14.95 (99.79*)
	HRV-A1	3.85	99.99
	AdV-5	4.56	99.99
	IFV-A H3N2	0.81	84.36
chitosan/AgNO_3_	HCoV-OC43	0.62 (4.80*)	35.94 (99.99*)
	HRV-A1	4.39	99.99
	AdV-5	0.24	41.86
	IFV-A H3N2	1.76	98.26

a R value and inhibition % calculated comparing the titers (FFU/cm^2^ or PFU/cm^2^) of the coatings with the titer (FFU/cm^2^ or PFU/cm^2^) of NO SAMPLE.

**Figure 10:**
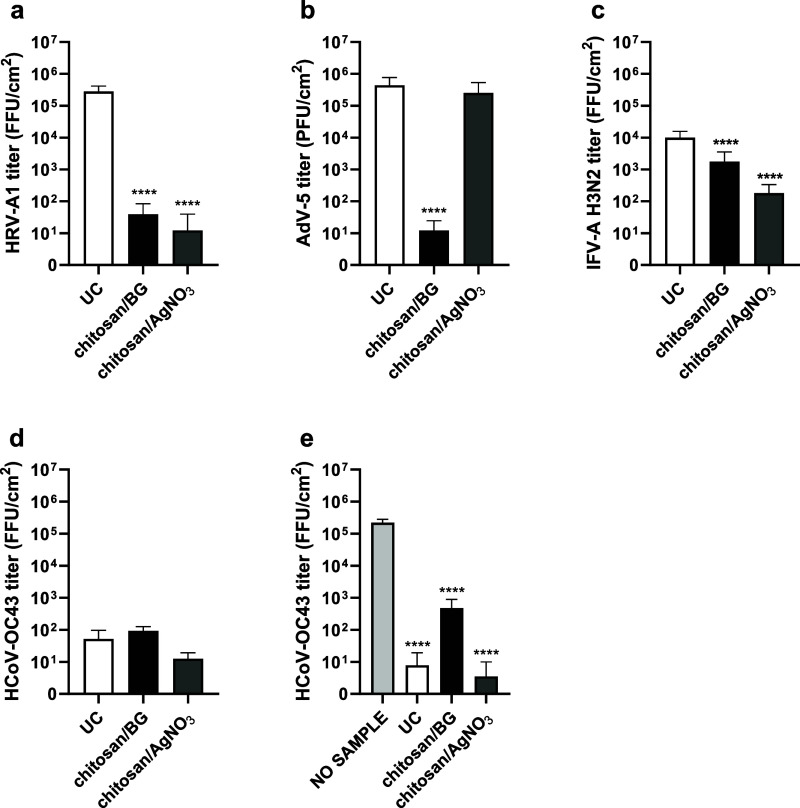
Chitosan/BG and chitosan/AgNO_3_ coatings exhibited broad-spectrum antiviral action against commonly circulating human respiratory viruses. Antiviral activity of chitosan/BG and chitosan/AgNO_3_ coatings was tested against (a)­HRV-A1, (b) AdV-5, (c) IFV-A H3N2, and (d) HCoV-OC43, following the ISO guidelines. After 24h at 25 °C of virus incubation with coated or uncoated metallic air filters, remaining virus particles were recovered and titrated. For (e) HCoV-OC43 the assay was repeated by adding a further control (NO SAMPLE) incubating the virus 24 h at 25 °C without any material. Viral titer is reported on the *Y*-axis and expressed as FFU or PFU per cm^2^ (FFU or PFU/cm^2^). Sample type is reported on the *X*-axis. UC, uncoated. **** *p*ANOVA <0.0001. Additional details of the assay protocol are described in the Materials and Methods section.

As previously stated, the uncoated substrate itself was shown to be active against HCoV-OC43. To better represent the antiviral activity of the coatings against HCoV-OC43, the *R* value and percentage of inhibition were calculated comparing the viral titer from coated samples with the no sample condition after incubation, instead of the uncoated sample as indicated in the ISO 21702:2019­(E).^21^ As depicted in Figure 10e, both coatings exerted a strong antiviral activity against HCoV-OC43, with an *R* value of 4.80 for chitosan/AgNO_3_ (inhibition %: 99.99) and of 2.67 for chitosan/BG (inhibition %: 99.79).

Overall, these data showed that chitosan/AgNO_3_ and chitosan/BG coatings are endowed with broad-spectrum antiviral activity against human respiratory viruses belonging to different families, reducing viral infectivity of both enveloped viruses (HCoV-OC43 and IFV-A H3N2) and naked viruses (HRV-A1 and AdV-5).

Subsequently, our interest was to investigate the mechanism of action of chitosan/AgNO_3_ and chitosan/BG coatings and to differentiate between a direct or indirect inactivation of viral particles. HCoV-OC43 and HRV-A1 were selected to assess the antiviral mechanism of action of the coated filters, due to the strongest inhibitory action reported for both coatings against the two viruses and the different virion structure.

In order to assess the antiviral mechanism of action, two different assays were performed. First, we conducted a drying test to investigate the possibility that the virus was inactivated by direct contact with the coated material. As shown in Table 7 and Figure 11a, a strong antiviral activity against HCoV-OC43 was observed in the drying test for both coatings, with an *R* value of 1.95 for chitosan/BG (inhibition % 98.88) and of 1.82 for chitosan/AgNO_3_ (inhibition % 98.49). Remarkably, no antiviral activity of the uncoated material against HCoV-OC43 was reported in this assay, showing an enhancement of the antiviral activity in the presence of the coatings, even after a short incubation time. Similarly, the two coated metallic filters demonstrated to be active against HRV-A1 in the drying test (Table 7 and Figure 11d), with an *R* value of 1.42 (inhibition % 96.17) and of 0.86 (inhibition %: 86.19) for chitosan/BG and chitosan/AgNO_3_, respectively.

**Table 7:** Numerical Results of the Drying Test

coating	virus	*R* value	inhibition %
chitosan/BG	HCoV-OC43	1.95	98.88
	HRV-A1	1.42	96.17
chitosan/AgNO_3_	HCoV-OC43	1.82	98.49
	HRV-A1	0.86	86.19

**Figure 11:**
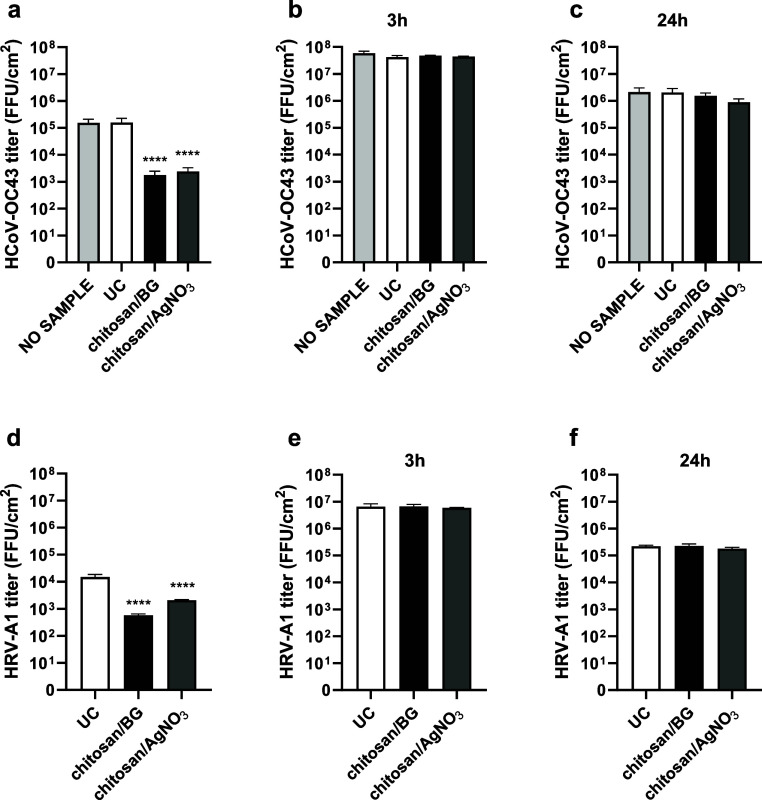
Chitosan/BG and chitosan/AgNO_3_ coatings exert antiviral activity by direct action on viral particles. The antiviral mechanism of action of coatings was investigated by a drying test and ion release test. The drying test was conducted against (a) HCoV-OC43 and (d) HRV-A1, by inoculating virus on coated metallic filter, or uncoated one as control, for 3 h under a biological hood until complete drying. The ion release test was performed against (b,c) HCoV-OC43 and (e,f) HRV-A1 by incubating the virus with ionic extracts for (b,e) 3 h or (c,f) 24 h. The viral titer is reported on the *Y*-axis and expressed as FFU per cm^2^ (FFU/cm^2^). Sample type is reported on the *X*-axis. UC, uncoated. **** pANOVA <0.0001. Additional details of the assay protocol are described in the Materials and Methods section.

Next, a second assay was performed, namely, ion release test, to assess a potential antiviral action of ions possibly released from the coatings. Results of the ion release test are reported in Table 8 and Figure 11b,e. As depicted, no antiviral activity was detected against HCoV-OC43 for both chitosan/BG (*R* value of −0.05, inhibition % −11.58) and chitosan/AgNO_3_ (*R* value of −0.02, inhibition % −3.77). A similar result was obtained against HRV-A1, since the two coatings showed no activity exhibiting an *R* value of −0.01 for chitosan/BG (inhibition %: −2.49) and 0.03 for chitosan/AgNO_3_ (inhibition %: 7.70). Afterward, a second ion release test was performed, extending the incubation time to 24 h, resembling the conditions of the antiviral test performed according to the ISO 21702:2019­(E).^21^ The assay was repeated to explore the possibility that ions could be released from the coatings and exert antiviral activity in a longer incubation time (Figure 11c,f). A slightly higher albeit still not significant antiviral activity against HCoV-OC43 was observed for both coatings, with an *R* value of 0.13 for chitosan/BG (inhibition %: 25.72) and 0.37 for chitosan/AgNO_3_ (inhibition %: 56.91). No antiviral action was reported against HRV-A1 for chitosan/BG (*R* value of −0.01, inhibition %: −2.86) and chitosan/AgNO_3_ (*R* value of 0.09, inhibition %: 19.02).

**Table 8:** Numerical Results of the Ion Release Test

coating	virus	*R* value (3 h)	inhibition % (3 h)	*R* value (24 h)	inhibition % (24 h)
chitosan/BG	HCoV-OC43	–0.05	–11.58	0.13	25.72
	HRV-A1	–0.01	–2.49	–0.01	–2.86
chitosan/AgNO_3_	HCoV-OC43	–0.02	–3.77	0.37	56.91
	HRV-A1	0.03	7.70	0.09	19.02

Data collected from the drying test and the ion release test showed that the antiviral effect exerted by the coated metallic air filters against HCoV-OC43 and HRV-A1 is mainly due to intrinsic antiviral activity, resulting from a direct contact of the viral particles with the coatings. Instead, ions released from the coatings partially contributed to the inhibitory effect. Similar assays were conducted by Luceri et al.^55^ on coated polymeric air filters, demonstrating an anticoronavirus action partially due to direct contact of virus with the coated material and partially mediated by the release of silver ions from the coating, confirming the reliability of our data.

The present study demonstrates that metallic air filters coated with chitosan/AgNO_3_ and chitosan/BG coatings exert a broad-spectrum antiviral activity against a panel of representatives of the most common human respiratory viruses following the ISO 21702:2019­(E) guidelines and that the tested coatings have a favorable cytocompatibility profile on tested cells.^21^ It is worth mentioning that the chitosan/BG coating was endowed with antiviral activity also against AdV-5, in contrast to chitosan/AgNO_3_. This last activity is particularly relevant, given the known high resistance of non-enveloped viruses to environmental conditions such as desiccation, detergents, pH and temperature.^56^ Notably, chitosan/BG also demonstrated stronger antiviral activity during the drying test against both HCoV-OC43 and HRV-A1. This data highlight the promising potential of this coating for future implementations.

## 4 Conclusion

The production of chitosan/AgNO_3_ and chitosan/BG coatings on metallic filters using EPD was successful. The incorporation of AgNO_3_ or BG in the chitosan coating matrix was confirmed by SEM, FTIR, and EDX analyses. The bubble point values for both coating types were in the range of the uncoated filters (128 ± 2 mbar) and suggest intact filtration capability after the application of the coatings. The antibacterial assay showed an antibacterial activity of >99% against *S. aureus* for both chitosan/AgNO_3_ and chitosan/BG coatings. Furthermore, both chitosan/AgNO_3_ and chitosan/BG-coated filters exhibited a lack of cytotoxicity and a wide antiviral activity against both enveloped (HCoV-OC43 and IFV-A H3N2) and non-enveloped (HRV-A1 and AdV-5) human respiratory viruses. It is interesting to note that chitosan/BG coatings outperformed chitosan/AgNO_3_ in their antiviral properties against AdV-5. The antiviral performance of the coated filters against HCoV-OC43 and HRV-A1 could be attributed to the direct contact of the viral particles with the coating material rather than to the activity of the released ions. This mechanistic insight into the direct inactivation of viral particles, independent of any contribution from ions released by the coatings, adds a significant layer of understanding to the current study and offers a deeper comprehension of the functional properties of the antiviral coating. Therefore, in addition to preserving the filtration efficiency of the base filter, the coating introduces an essential functional enhancement by actively inactivating microorganisms that are captured on the filter surface. Unlike conventional filters that merely retain viable pathogens, chitosan/BG and chitosan/AgNO_3_ coatings contribute to a significant reduction in the risk of microbial survival, proliferation, and potential rerelease into the air stream. This antimicrobial action enhances the overall hygienic performance of the filter, particularly in enclosed or recirculating air environments, and may help to extend filter service intervals by reducing biological fouling. Future work should focus on the investigation of the material–virus interactions concerning some intrinsic filter properties, such as pore size, degree of porosity, mechanical properties, electrical charge, wettability, etc. Considering the targeted applications for air filtration and the associated risk of filter pore clogging due to the accumulation of contaminants, a more extensive investigation on the long-term antibacterial and antiviral activities of chitosan/BG and chitosan/AgNO_3_ coatings needs to be performed. In particular, future experiments will include long-term tests (up to one year) under simulated indoor conditions, following ISO guidelines, to assess coating durability and efficacy. A functional prototype will be developed to test viral aerosol reduction under dynamic airflow. Additionally, microbial reduction will be evaluated in real-world environments such as hospitals and public spaces. These studies will offer key insights into the practical performance and robustness of the coatings in air filtration applications. Moreover, additional studies are required to verify the suitability of the proposed coating material and fabrication technique for up-scaled industrial production. Still, taking into account the adequate filtration capability, the excellent antibacterial properties, and the enhanced antiviral activity, the so-produced chitosan/BG and chitosan/AgNO_3_ EPD coatings could provide a promising solution to improve the air quality in a range of daily settings (e.g., healthcare facilities, vehicle cabins, public transport, etc.), and thus the presented coating technology addresses some of the challenges associated with air filtration.
